# Sleep duration is associated with white matter microstructure and cognitive performance in healthy adults

**DOI:** 10.1002/hbm.25132

**Published:** 2020-07-10

**Authors:** Pascal Grumbach, Nils Opel, Stella Martin, Susanne Meinert, Elisabeth J. Leehr, Ronny Redlich, Verena Enneking, Janik Goltermann, Bernhard T. Baune, Udo Dannlowski, Jonathan Repple

**Affiliations:** ^1^ Department of Psychiatry University of Münster Münster Germany; ^2^ Department of Economics University of Münster Münster Germany; ^3^ Department of Psychiatry Melbourne Medical School, The University of Melbourne Melbourne Victoria Australia; ^4^ The Florey Institute of Neuroscience and Mental Health The University of Melbourne Parkville Victoria Australia

**Keywords:** cognitive performance, DTI, fractional anisotropy, HCP, sleep quality

## Abstract

Reduced sleep duration and sleep deprivation have been associated with cognitive impairment as well as decreased white matter integrity as reported by experimental studies. However, it is largely unknown whether differences in sleep duration and sleep quality might affect microstructural white matter and cognition. Therefore, the present study aims to examine the cross‐sectional relationship between sleep duration, sleep quality, and cognitive performance in a naturalistic study design, by focusing on the association with white matter integrity in a large sample of healthy, young adults. To address this, 1,065 participants, taken from the publicly available sample of the Human Connectome Project, underwent diffusion tensor imaging. Moreover, broad cognitive performance measures (NIH Cognition Toolbox) and sleep duration and quality (Pittsburgh Sleep Quality Index) were assessed. The results revealed a significant positive association between sleep duration and overall cognitive performance. Shorter sleep duration significantly correlated with fractional anisotropy (FA) reductions in the left superior longitudinal fasciculus (SLF). In turn, FA in this tract was related to measures of cognitive performance and was shown to significantly mediate the association of sleep duration and cognition. For cognition only, associations shift to a negative association of sleep duration and cognition for participants sleeping more than 8 hr a day. Investigations into subjective sleep quality showed no such associations. The present study showed that real‐world differences in sleep duration, but not subjective sleep quality are related to cognitive performance measures and white matter integrity in the SLF in healthy, young adults.

## Graphical abstract

We investigated in 1,065 participants, taken from the publicly available sample of the Human Connectome Project, the association of sleep duration and quality with brain white matter microstructure and cognitive performance. The results revealed a significant positive association between sleep duration and overall cognitive performance. Shorter sleep duration significantly correlated with FA reductions in the left superior longitudinal fasciculus (SLF). In turn, FA in this tract was related to measures of cognitive performance and was shown to significantly mediate the association of sleep duration and cognition.

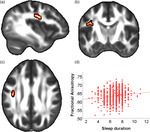

## 1 INTRODUCTION

Sleep is an important evolutionary conserved process, which is essential for human well‐being (Elvsåshagen et al., 2015). There is no evidence that humans or animals can renounce sleep without detrimental consequences (Cirelli & Tononi, 2008). Decreased sleep duration as well as poor subjective sleep quality have both been identified as important risk factors for several mental and somatic disorders and are common in western societies (Ohayon, 2002). According to an international survey, nearly a third of the interviewed western European individuals reported any sleep problem, respectively more than 50% in the United States of America (Léger, Poursain, Neubauer, & Uchiyama, 2008; Penzel, Peter, & Peter, 2005). In turn, sleep problems are associated with subsequent reduced well‐being, accidents, alcoholism, health problems, depression, and cognitive impairment (Ohayon, 2002).

The physiological and neuropsychological research of sleep and its link to cognition has been intensified in the last decade (Deak & Stickgold, 2010; Goel, Rao, Durmer, & Dinges, 2009; Lim & Dinges, 2010): Experimental induced sleep deprivation affects cognitive performance with most robust results for executive function (Van Dongen, Maislin, Mullington, & Dinges, 2003), working memory (Chee & Choo, 2004), verbal learning and language (Harrison & Horne, 1998), and attention (Lim & Dinges, 2008). This emphasizes the association of sleep loss with those cognitive tasks that especially require prefrontal activity (Wu et al., 2006). Studies examining the effect of sleep duration and sleep quality on cognitive performance show similar results with significantly decreased verbal and working memory performance (Lo, Groeger, Cheng, Dijk, & Chee, 2016; Nebes, Buysse, Halligan, Houck, & Monk, 2009), although the results are more heterogeneous for subjective sleep quality (Cavuoto et al., 2016; Saint Martin, Sforza, Barthélémy, Thomas‐Anterion, & Roche, 2012). Furthermore, there is evidence that not only decreased sleep duration, but also prolonged sleep duration is linked to poorer cognitive performance suggesting an inverse U‐shaped association between sleep quantity and cognition (Lo et al., 2016).

While the relation of short and long‐lasting sleep to cognitive performance is widely investigated, the underlying alterations in brain physiology and structural connectivity have only recently been further examined. For instance, shorter sleep duration was associated with decreased fractional anisotropy (FA), a neuroimaging marker of microstructural white matter integrity, in occipital and parietal regions in elderly subjects (Yaffe et al., 2016). Another cross‐sectional study showed that patients with primary insomnia had significantly lower FA values compared to healthy controls in white matter tracts such as the superior longitudinal fasciculus (SLF), which contains the primary axons connecting the fronto‐parietal pathway (Li et al., 2016). Functional responsiveness of this fronto‐parietal attention system and microstructural properties of the SLF additionally seem to be associated with the ability to sustain attention during sleep deprivation (indicated as the cognitive vulnerability to insufficient sleep), drawing a link to white matter and cognitive performance (Cui et al., 2015; Rocklage, Williams, Pacheco, & Schnyer, 2009). Interestingly, an experimental study found that a day of waking (14 hr after a night of normal sleep) is associated with increased FA in white matter tracts including the right SLF, whereas sleep deprivation the night after is associated with widespread FA decreases. The authors concluded that the human brain shows circadian plasticity and that microstructural changes can be found even over hours to days (Elvsåshagen et al., 2015).

Although there are two studies which found significant positive associations between sleep quality and white matter integrity (Khalsa et al., 2017; Sexton et al., 2017), another study showed shorter sleep duration and better sleep quality to be associated with lower mean diffusivity, another white matter microstructure marker, in the prefrontal cortex and right hippocampus (Takeuchi et al., 2018). This demonstrates the inconsistency within the literature. As white matter integrity has been linked to cognitive performance (Bennett & Madden, 2014; Kochunov et al., 2010; Opel et al., 2019; Repple et al., 2019), investigations into the associations with brain structural connectivity could shed light on the sleep‐cognition relationship.

Subjective sleep quality, sleep duration, cognitive performance, and white matter microstructure have not yet been investigated in a large sample of healthy, young adults. More precisely, we know that experimental sleep deprivation leads to decreased FA and impaired cognition, but it is largely unknown whether interindividual differences in sleep duration or sleep quality are associated with decreased microstructural white matter integrity and cognition. To this end, sleep is not experimentally deprived in our study, but differences of real‐world sleep habits are taken from a large, representative sample.

To address this currently unresolved question, we aimed to investigate associations of sleep duration and subjective sleep quality with cognitive performance and white matter microstructure in a well‐powered sample of healthy, young adults. We hypothesized that (a) short sleep duration and poor quality are associated with reduced cognitive performance with strongest effect sizes for sleep duration, that (b) short sleep duration is associated with lower FA in the SLF, and that (c) reduced FA in this fiber tract positively correlates with cognitive performance and that it mediates the association of sleep duration and cognition.

## 2 MATERIAL AND METHODS

### 2.1 Participants

The following methods regarding the human connectome project (HCP) dataset have been extensively described in our previous work (Repple, Karliczek, et al., 2019). In brief, the data was taken from the open‐access WU‐Minn HCP 1200 Subjects Data Release (Van Essen et al., 2013). The WU‐Minn HCP aims for a better understanding of human brain connectivity and function in a population of 1,206 healthy adults. Individuals with neurodevelopmental disorders (e.g., autism), neuropsychiatric disorders (e.g., schizophrenia or depression), neurologic disorders (e.g., Parkinson's disease), diabetes, or high blood pressure were excluded, because of their negative impact on brain structure (Van Essen et al., 2013). In the present study, analyses were made with the maximum number of available data for each analysis. The respective n for each analysis will be reported.

The mean age of the 1,206 participants was 28.8 years (ages 22–37, *SD* = 3.7), 54.4% of them were female. The participants were primarily born in Missouri. Additional recruiting efforts were undertaken to ensure that individuals widely correspond to the ethnic and racial composition of the U.S. population. After that, participants visited the Washington University twice for fixed imaging procedures (structural MRI, resting‐state fMRI, task fMRI, and DTI) and extensive behavioral assessment (Van Essen et al., 2013).

### 2.2 Sleep measures

Sleep measures were available for *n* = 1,206 subjects. The Pittsburgh sleep quality index (PSQI) is a self‐report questionnaire that assesses sleep quality and duration during the previous month (Buysse, Reynolds, Monk, Berman, & Kupfer, 1989; for description of all PSQI subcomponents, please see Supplementary Material S1). Sleep duration was numerically assessed with the question “*During the past month*, *how many hours of actual sleep did you get at night? (This may be different than the number of hours you spend in bed)”*, whereas subjective sleep quality was assessed with the question “*During the past month*, *how would you rate your sleep quality overall* on a scale from *very good* (0) to *very bad* (3)”. Overall test–retest reliability was 0.53 for sleep quality and 0.80 for sleep duration in one psychometric evaluation of the PSQI in primary insomnia (*p* < .001). Moreover, validity analyses between sleep duration and sleep log data showed a high correlation (*r* = .81, *p* < .001) (Backhaus, Junghanns, Broocks, Riemann, & Hohagen, 2002).

### 2.3 Cognitive performance

Cognitive measures were available for *n* = 1,187 subjects. All cognitive subdomains of the NIH Toolbox Cognition Battery were included in the present study. Cognition is one of four domains assessed by the NIH Toolbox, whereas the Cognition Battery consists of seven subscales (Flanker Inhibitory, Picture Sequence Memory, List Sorting and Pattern Comparison, Picture Vocabulary and Oral Reading Recognition, Dimensional Change Card Sort) with an overall duration of 30 min (Weintraub et al., 2013). The Cognitive Function Composite Score constitutes global cognitive performance and is calculated as the average of all subscores. Higher scores reflect better cognitive performance. All scores underwent standardization with 100 as the average performance and ±15 points representing one *SD* above or below. For further information about the respective measures, see Supplementary Material S2.

### 2.4 DTI data acquisition

DTI data were available for *n* = 1,065 subjects. Data from all HCP participants was acquired on a customized Siemens 3T “Connectome Skyra” at Washington University by using a standard 32‐channel Siemens receive head coil and a “body” transmission coil. This was specifically designed by Siemens for the smaller space available using the special gradients of the WU‐Minn and MGH‐UCLA Connectome scanners (Van Essen et al., 2013).

A full diffusion MRI session includes six runs (each ~9 min and 50 s), representing three different gradient tables, with each table acquired once with right‐to‐left and left‐to‐right phase encoding polarities, respectively. Each gradient table includes ~90 diffusion weighting directions plus 6 b = 0 acquisitions distributed throughout each run. Diffusion weighting consisted of 3 shells of b = 1,000, 2000, and 3,000 s/mm^2^ interspersed with an approximately equal number of acquisitions on each shell within each run (Sequence: Spin‐echo EPI, TR 5520 ms, TE 89,5 ms, flip angle 78 deg, refocusing flip angle 160 deg, FOV 210 × 180 (RO × PE), matrix 168 × 144 (RO × PE), slice thickness 1.255 mm, 111 slices, 1.25 mm isotropic voxels, multiband factor 3, echo spacing 0.78 ms, BW 1488 Hz/Px, phase partial fourier 6/8, b‐values 1,000, 2000, 3,000 s/mm^2^).

### 2.5 DTI data preprocessing and analysis

Diffusion data accessible from the HCP were preprocessed with their MR Diffusion Pipeline (Glasser et al., 2013), which normalizes the b0 image intensity across runs; removes EPI distortions, eddy‐current‐induced distortions, and subject motion; corrects for gradient‐nonlinearities; registers the diffusion data with the structural; brings it into 1.25 mm structural space; and masks the data with the final brain mask: (a) Basic preprocessing: Intensity normalization across runs, preparation for later modules. (b) “TOPUP” algorithm for EPI distortion correction. (c) “EDDY” algorithm for eddy current and motion correction. (d) Gradient nonlinearity correction, calculation of gradient bvalue/bvector deviation. (e) Registration of mean b0 to native volume T1w with FLIRT BBR + bbregister and transformation of diffusion data, gradient deviation, and gradient directions to 1.25 mm structural space. The brain mask is based on FreeSurfer segmentation.

A well‐established analysis method for DTI imaging is Tract‐based spatial statistics (TBSS; Smith et al., 2006) and was described in a previous study (Repple et al., 2018): Standard TBSS preprocessing was performed (Smith et al., 2006): The FA images were registered to the FMRIB58 FA template and averaged to create a mean FA image. A WM skeleton was created with an FA threshold of 0.2 and overlaid onto each subject's registered FA image. Individual FA values were warped onto this mean skeleton mask by searching perpendicular from the skeleton for maximum FA values.

We used the nonparametric permutation testing implemented in FSL's “randomize” with 5,000 permutations, to test for statistical significance. Threshold‐free cluster enhancement (TFCE; Smith & Nichols, 2009), a previously employed method (Repple et al., 2019; Repple, Karliczek, et al., 2019) to correct for multiple comparisons, allows estimation of cluster sizes corrected for the family‐wise error (FWE; *p* < .05, 5,000 permutations). MNI coordinates and cluster size at peak voxel were derived with FSL Cluster and the corresponding WM tract retrieved from the ICBM‐DTI‐81 white‐matter atlas (Pekar et al., 2007; Repple et al., 2019).

### 2.6 Statistical analyses

Statistical analyses were performed within SPSS (IBM Version 25).

1. The correlation analysis between sleep duration and sleep quality (spearman correlation because of ordinal scale) on one side and global cognitive performance on the other side was examined correcting for age and sex. Additionally, exploratory correlational analyses between sleep measures and all available cognitive measures were performed, again correcting for age and sex.
2. We investigated the association between differences in sleep measures and alterations of FA. Therefore, linear effects between sleep duration or sleep quality and FA in the superior longitudinal fascicles were tested within FSL each in a specific general linear model. Additionally, exploratory analyses were repeated across the whole brain. All analyses were corrected for age, sex, and intracranial volume.
3. To assess if FA values from the analysis above are associated with cognition, a correlation analysis was performed between extracted FA values (from regions that significantly correlated with sleep duration or sleep quality) and the global cognition score, again correcting for age and sex. As already described in hypothesis 1, exploratory correlational analyses between FA and cognitive subdomains were additionally assessed.
4. To test for an effect of sleep duration or sleep quality on cognitive performance through microstructural WM, we performed a mediation analysis with the sleep measure as predictor variable (X), extracted mean FA from the sleep duration analysis (hypothesis 2) as mediator (M) and global cognitive performance as outcome variable (Y) with age and sex as covariates. The procedure has been extensively described in our previous study (Opel et al., 2019). Briefly, a bootstrapping approach as implemented in the SPSS macro PROCESS was applied (http://www.processmacro.org) which has been demonstrated to provide reliable results in neuroimaging research (Fontana, Eagon, Trujillo, Scherer, & Klein, 2007; Frodl et al., 2012; Opel et al., 2017). PROCESS estimates direct and indirect effects between a defined set of variables by applying an ordinary least squares path analytic framework. Inference of indirect (mediated) effects is assessed through bootstrap confidence intervals. Significance of indirect effects is assumed if the 95% confidence interval (95% CI) does not include zero. The number of bootstrap iterations was set to *n* = 5,000. Unstandardized regression coefficients (coeff) and standard errors (*SE*) are presented for each effect.
5. We performed several post hoc exploratory analyses: (a) There is evidence that not only decreased sleep duration, but also prolonged sleep duration is linked to poorer cognitive performance suggesting an inverse U‐shaped association between sleep quantity and cognition (Lo et al., 2016). To test this, we specified a quadratic model (Supplementary Material S3) as well as further analyses in subsamples formed according to the level of sleep duration (short sleep duration (0–5 hr), moderate sleep duration (5.5–8 hr), prolonged sleep duration (8.5–12 hr) (Watson et al., 2015); Supplementary Material S4). (b) Furthermore, several studies (Cournot et al., 2006; Meyer, Wall, Larson, Laska, & Neumark‐Sztainer, 2012; Repple et al., 2018) as well as our data (Supplementary Material S5) revealed associations between the Body Mass Index (BMI) and sleep duration, cognitive function and white matter microstructure within the SLF. Therefore, we repeated the analyses described above with BMI as an additional control variable (Supplementary Material S6). (c) To further disentangle different aspects of sleep quantity and quality, post hoc exploratory analyses with all PSQI subcomponents were correlated with available cognitive measures and FA (for description of all PSQI subscores see Supplementary Material S1, for correlation matrix of all PSQI subcomponents, see Supplementary Material S7, for nonparametric tests of all PSQI subscores with cognition and FA see Supplementary Material S8). (d) To test whether the observed associations differed in strength depending on the age of the participants, interaction analyses with sleep duration*age interactions terms were performed (for details see Supplementary Material S9).

## 3 RESULTS

### 3.1 Sleep measures and cognitive performance

Statistical analysis revealed a small, but significant, positive correlation between sleep duration and the global cognition score (*df* = 1,183, *r* = .097, *p* = .001) while correcting for age and sex. Significant positive correlations between sleep duration and cognitive sub‐scores could be found for picture vocabulary test and oral reading recognition test (*df* = 1,183, *r* = .116, *p* < .001 and *r* = .135, *p* < .001). Correlational analyses showed no significant association of subjective sleep quality with any cognitive scale. For results of all respective analyses, please see Table 1. For nonparametric sleep‐cognition associations showing consistent results, see Supplementary Material S8.

**TABLE 1:** Correlation of sleep quality, sleep duration and fractional anisotropy with cognitive subscores

	Sleep quality	Sleep duration	Fractional anisotropy
NIH total cognition score			
Pearson's r	.002	**.097** ^***^	**.089** ^**^
*p*‐value	.938	**<.001**	**.004**
*df*	1,183	**1,183**	**1,046**
Flanker inhibitory control and attention test: Executive function			
Pearson's r	.005	.015	.010
*p*‐value	.863	.597	.754
*df*	1,183	1,183	1,046
Picture sequence memory: (nonverbal) episodic memory			
Pearson's r	−.007	.052	**.064** ^*^
*p*‐value	.813	.073	**.038**
*df*	1,183	1,183	**1,046**
List sorting working memory test			
Pearson's r	.028	.041	**.088** ^**^
*p*‐value	.330	.162	**.005**
*df*	1,183	1,183	**1,046**
Picture vocabulary test: Vocabulary knowledge			
Pearson's r	−.005	**.116** ^***^	.038
*p*‐value	.853	**<.001**	.214
*df*	1,183	**1,183**	1,046
Oral reading recognition test: Reading decoding skills			
Pearson's r	−.014	**.135** ^***^	.060
*p*‐value	.640	**<.001**	.053
*df*	1,183	**1,183**	1,046
Dimensional change card sort test: Executive function and cognitive flexibility			
Pearson's r	.018	.049	.055
*p*‐value	.539	.092	.073
*df*	1,183	1,183	1,046
Pattern comparison processing speed test			
Pearson's r	−.001	−.003	.055
*p*‐value	.967	.904	.077
*df*	1,183	1,183	1,046

*Note:* Sleep duration: Hours of actual sleep at night; Sleep quality: Overall rating of subjective sleep quality on a 0–3 scale; SLF: superior longitudinal fasciculus; FA: fractional anisotropy, mean value extracted from the FA‐global cognition association results mask; Pearson's r = Pearson correlation coefficient; *p*‐value = value determining statistical significance, *** *p* < .001; ** *p* < .01; * *p* < .05, values < .05 in bold font; *df* = degrees of freedom; for more information on the cognitive subscores see Supplementary Material S2.

### 3.2 Sleep measures and white matter microstructure

**FIGURE 1:**
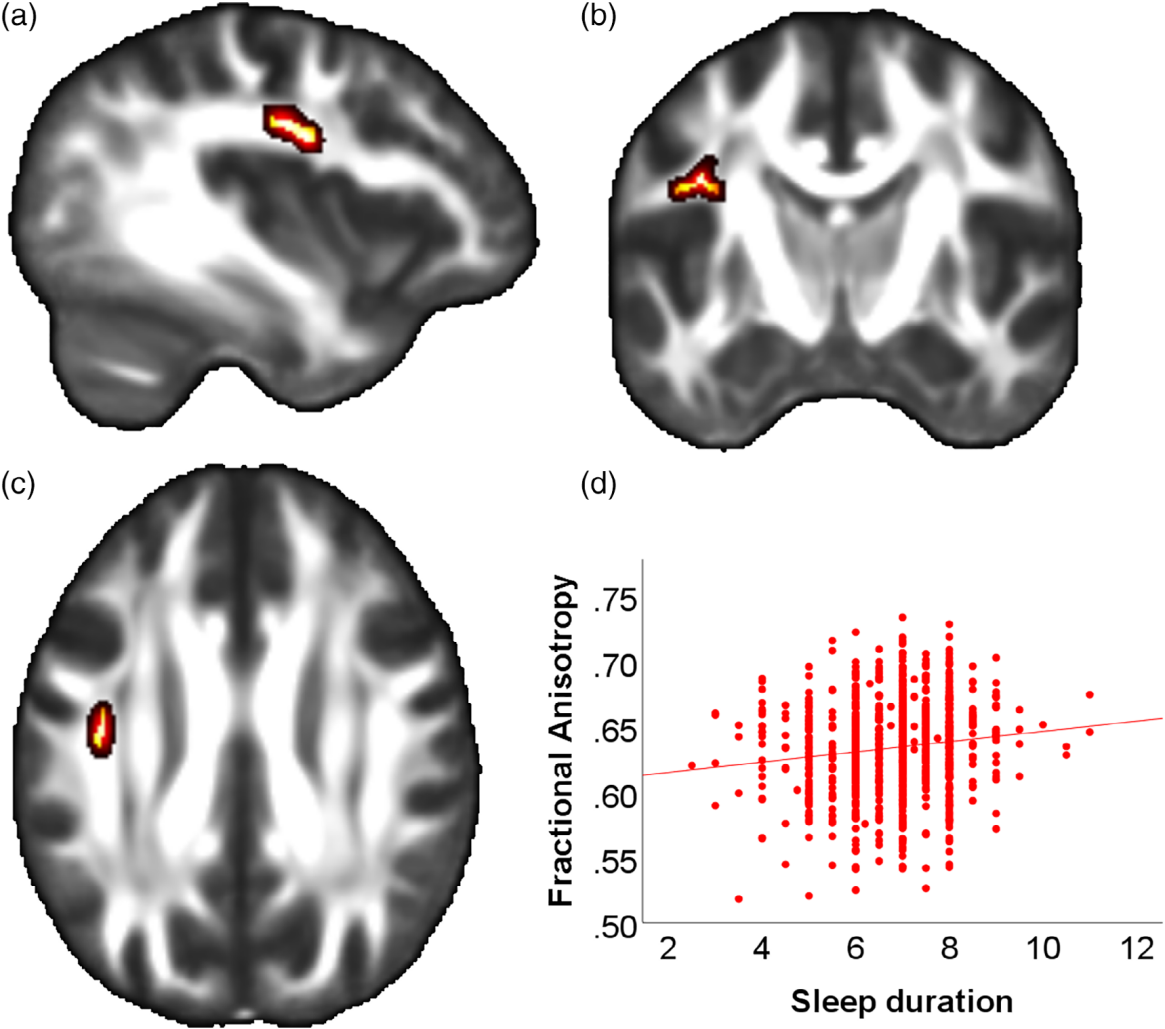
Association of sleep duration and fractional anisotropy in the left superior longitudinal fasciscle. (a) Sagittal slice with x = −38 (MNI) of a mean FA map. Red‐yellow areas represent voxels (using FSL's “fill” command for better visualization), where a significant positive association between sleep duration and Fractional Anisotropy was detected (left superior longitudinal fascicle, p_FWE_ < .05); (b) Coronal slice (y = −10) (c) Axial slice (z = 29); (d) Scatterplot showing the association sleep duration and extracted mean FA values from all significant voxels of the corresponding TBSS analysis

Complete PSQI and DTI data was available for *n* = 1,065 subjects. The ROI analysis revealed a positive association of sleep duration with FA in the left superior longitudinal fasciculus (SLF; *p*_*fwe*_ = .006, 202 voxel, peak MNI coordinates: −35, −9, 24, Figure 1), whereas sleep quality showed no such association (*p*_*fwe*_ = .693).

Exploratory whole‐brain analysis showed a trend toward a significant positive association between sleep duration and FA in several white matter tracts including the SLF, the corticospinal tract, the thalamic radiation and the internal capsule (*p*_*fwe*_ = .056, for further details see Supplementary Material 10). No significant association between subjective sleep quality and FA was found (*p*_*fwe*_ = .497).

### 3.3 White matter microstructure and cognitive performance

Extracted FA from the SLF was associated with global cognition (*df* = 1,046, *r* = .089, *p* = .004). Moreover, additional correlational analyses showed significant but small associations between extracted FA in the SLF and cognitive performance in the picture sequence memory test (episodic memory; *df* = 1,046, *r* = .064, *p* = .038) as well as the list sorting working memory test (working memory; *df* = 1,046, *r* = .088, *p* = .005). For an overview of all analyses, see Table 1.

### 3.4 Mediation analyses

In line with analyses steps 3.1–3.3 the mediation model confirmed a significant positive association between sleep duration and FA (coeff = .004, SE = .001, 95% CI = .002 to .006, t = 4.142, *p* < .001) as well as a significant association between FA and global cognitive performance (coeff = 32.995, SE = 12.936, 95% CI = 7.761 to 58.379, t = 2.551, *p* = .011). The mediation model furthermore yielded a significant positive indirect (mediated) effect of sleep duration on cognitive performance through FA (indirect effect: coeff = .128, SE = .060, 95% CI = .024 to .259) with a persistent direct effect (coeff = .990, SE = .394, 95% CI = .217 to 1.763, t = 2.514, *p* = .012), suggesting a partial mediation.

### 3.5 Exploratory analyses

**(**a) In the quadratic regression model with global cognition as the dependent variable, a significant effect of the squared sleep duration regressor (ß = −.577; *p* = .001, for full regression analysis results see Supplementary Material S3) was estimated, suggesting an inverse U‐shaped relationship of sleep duration and cognitive performance (see Supplementary Figure S1 in Supplementary Material S3 for illustration). In line with that, subgroup analyses based on sleep duration showed a positive association of sleep duration and cognition in the moderate sleep duration group (r = .074; *p* = .028; df = 880) and a negative association in the prolonged sleep duration group (r = −.393; *p* = .003; df = 53), for all subgroup results see Supplementary Material S4. (b) After adding BMI to our control variables (beside age and sex), all prior significant associations remained significant with only little decrease of effect sizes (for an overview see Supplementary Material S6). (c) Nonparametric post hoc exploratory analyses of all PSQI subscores revealed negative associations of total cognition with the global PSQI score (r = −.102; *p* < .001; *df* = 1,187) as well as negative associations with the PSQI subcomponents sleep latency, habitual sleep efficiency, sleep disturbance, and sleep duration (Supplementary Material 8). Furthermore, only the global PSQI score (r = −.063; *p* = .040; *df* = 1,065) and sleep duration (r = −.112; p < .001; *df* = 1,065) were in turn correlated with extracted FA in the SLF. (d) We could not detect any sleep duration * age interactions (see Supplementary Material 9).

## 4 DISCUSSION

To our knowledge, this is the first study to investigate the relationship between subjective sleep quality, sleep quantity, cognitive performance, and white matter microstructure in a well‐powered sample of healthy, young adults. Our findings demonstrate that reported sleep duration, but not subjective sleep quality is associated with both cognitive performance, especially in language subdomains, and white matter integrity of the SLF irrespective of age, sex, or BMI. Moreover, the mediation analysis showed that white matter microstructure mediated the association of sleep duration and cognitive performance. Taken together, our results suggest that cognition and white matter integrity are not only affected by experimental sleep deprivation but are also associated with natural differences in habitual sleep duration.

The findings of the present study are in line with other studies, which showed that cognitive performance and white matter are associated with sleep duration. A previous study demonstrated that only objective total sleep time, but not subjective sleep indices predicted memory performance in community‐based older adults (Cavuoto et al., 2016). In contrast, other studies demonstrate that neither subjective nor objective measures of sleep (measured with the PSQI) are associated with cognitive performance in elderly participants (Blackwell et al., 2011; Saint Martin et al., 2012). We did not find any interactions with age in our analyses, but as the mean age is comparably low (mean age = 28.8) and the age range (22–37 years) is restricted in our sample, future studies with larger samples across the whole lifespan should investigate age‐related differences in the association of sleep duration and brain structure and function to address these heterogeneous findings. Previous findings of a nonlinear (quadratic) relationship between sleep duration and cognition could be replicated with the present sample. Our exploratory analyses revealed a negative U‐shaped association of sleep duration with cognition. Nonetheless, the coefficient on the linear sleep duration regressor remained significant in this model as well, suggesting a stronger association of reduced sleep duration with cognition compared to increased sleep duration. In line with that, statistical analyses in which our sample was divided into subsamples based on sleep duration validate the finding of an inverse U‐shaped association. Although increased sleep duration is negatively associated with cognition, the association of sleep duration with cognition is positive for short and moderate (recommended (Hirshkowitz et al., 2015)) sleep durations. As there is no clear biological hypothesis linking prolonged sleep duration and cognition yet (Marshall & Stranges, 2010), questions remain whether this cross‐sectional observation of prolonged sleep duration and impaired cognition in this comparatively small group (*n* = 55) could also be the result of comorbid risk factors (Patel, Malhotra, Gottlieb, White, & Hu, 2006). In contrast, our nonlinear analysis revealed no significant association of the coefficient estimate for the squared sleep duration regressor with fractional anisotropy in the SLF.

However, our results from the linear analyses demonstrate a positive association of sleep duration with FA in the left SLF and a trend‐level positive association of sleep duration with FA on a whole‐brain approach in several white matter tracts including the bilateral SLF. The white matter integrity of the SLF has been associated with abnormal sleep patterns in several other studies (Elvsåshagen et al., 2015; Li et al., 2016; Rocklage et al., 2009). The SLF is a major association fiber tract connecting the frontal cortex with the parietal and temporal lobe (Wang et al., 2016) and therefore plays an important role in language function and working memory. Thus, shorter sleep duration might possibly affect white matter integrity of the SLF that in turn is likely to result in impaired cognition, for example, working and episodic memory performance (Karlsgodt et al., 2008). This idea is supported by the mediation analyses, but as all data are cross‐sectional, this speculation should be investigated by future longitudinal or experimental studies. Our results, showing sleep quantity rather than sleep quality to be associated with white matter microstructure, are in line with several neurobiological investigations into effects of sleep on the brain. Oligodendrocyte precursor cells, for example, are important for the myelination of the brain (Bellesi et al., 2013). Their proliferation is negatively affected by wake and doubles during sleep. Thus, shorter sleep duration affects white matter microstructure on the cellular level by impairing oligodendrocyte physiology which in turn lead to cell dysfunctions that can reduce axonal integrity (Bellesi, 2015). Additionally, modest sleep loss and chronic sleep deprivation have been associated with increased concentrations of the proinflammatory cytokines IL‐6 and TNFα and significant impairment of glucose metabolism (Spiegel, Leproult, & Van Cauter, 1999; Vgontzas et al., 2004). Dysregulation of proinflammatory cytokines is linked to impairment of myelination and can lead to apoptosis (McEwen, 2006) and could therefore also cause disruption of white matter integrity.

Inconsistency of the literature regarding sleep quality might be a result of methodological differences regarding sleep measures. For instance, in one study (Takeuchi et al., 2018) the authors used mean diffusivity, a rather unspecific marker of overall diffusion in a specific voxel, to measure microstructural properties of the brain, which diminishes the comparability with the present study. Other studies used the global PSQI score as a measure for sleep quality (Khalsa et al., 2017; Sexton et al., 2017). However, the PSQI global score consists of seven distinct subcomponents (Buysse et al., 1989). Therefore, it could be possible that significant sleep quality‐FA associations in these studies were driven by one of these subcomponents (e.g., sleep duration). This motivated us to investigate the association of sleep duration and sleep quality with cognition and white matter microstructure separately by using the respective subcomponents of sleep duration and subjective sleep quality. Results of exploratory nonparametric analyses of all PSQI subscores revealed negative associations not only of sleep duration, but also of the global PSQI score, sleep latency, habitual sleep efficiency and sleep disturbance with global cognition. While results should be interpreted carefully because nonparametric tests cannot account for covariates as age and sex, these subscores are most strongly associated with sleep duration. This corroborates the notion that rather sleep duration than subjective sleep quality is driving the observed associations.

Although the present study has several strengths including a wide range of cognitive measures and the large and healthy, young sample, there are also some limitations. One major limitation is the cross‐sectional nature of all associations that does not permit any strong causal interpretations. Further longitudinal studies are needed to clarify the causal relationship between sleep duration, cognitive performance, and white matter integrity. Until then, the present results should be interpreted with caution. Moreover, the individual reason for the variation in sleep duration is not assessed in this study. This makes it challenging to make strong recommendations based on this real‐world data. For instance, it is largely known that individuals who report sleep problems systematically underestimate their actual sleep duration (Van Den Berg et al., 2008). Future studies (including those with experimental designs) should focus on more objective sleep measures, for example, laboratory measurements.

To conclude, our findings suggest an association of short and long sleep durations with cognitive performance and an association of shorter sleep duration with decreased integrity of white matter microstructure. Considering the growing incidence of sleep problems in western societies, the present study points toward the possible importance of recommended nighttime sleep for healthy brain structure and function.

## DISCLOSURE OF INTEREST

All authors declare no conflict of interest. All authors have approved the final article.

## AUTHOR CONTRIBUTIONS

Pascal Grumbach and Jonathan Repple made substantial contributions to the conception, design of the work, and drafted the work. Nils Opel, Stella Martin, Ronny Redlich, Elisabeth J. Leehr, Janik Goltermann, Verena Enneking made substantial contributions to the analysis of this work and the interpretation of data. Bernhard T. Baune, Udo Dannlowski have substantially revised the work. All authors approved the submitted version and agreed both to be personally accountable for the author's own contributions and that questions related to the accuracy or integrity of any part of the work are appropriately investigated.

## Supporting information


**Appendix** **S1.** Supporting Information.

## Data Availability

In brief, the data were taken from the open‐access WU‐Minn HCP 1200 Subjects Data Release [Van Essen et al., 2013].
