# Supplementary material for: Sleep duration is associated with white matter microstructure and cognitive performance in healthy adults
Source: Hum Brain Mapp. 2020 Jul 10;41(15):4397–405. doi: 10.1002/hbm.25132 (PMC7502839; doi:10.1002/hbm.25132)
Supplement: Supplementary file 1 — Appendix S1. Supporting Information. [file HBM-41-4397-s001.docx]

**Supplementary Material 1: Subcomponents of the Pittsburgh Sleep Quality Index**

| **Subcomponent** | **Description** | **Score** | **Reference** |
| --- | --- | --- | --- |
| Subjective Sleep Quality | Overall subjective sleep quality | Very good (0)  Fairly good (1)  Fairly bad (2)  Very bad (3) | [Buysse, D.J., Reynolds, C.F., Monk, T.H., Berman, S.R., & Kupfer, 1989]  (Taken from the original questionnaire and the evaluation chart) |
| Sleep Latency | (1) Amount of time which it has usually take to fall asleep each night  (2) Frequency of such sleep induction problems | **Sum of both questions (0-3):**  0: ≤ 15 min  1: 16-30 min  2: 31-60 min  3: ≥ 60 min  Not during the past month (0)  Less than once a week (1)  Once or twice a week (2)  Three or more times a week (3) |  |
| Sleep Duration | Hours of actual sleep each night | 0: > 7  1: 6-7  2: 5-6  3: < 5 |  |
| Habitual sleep efficiency | (Number of hours slept/Number of hours spent in bed) x 100 (in %) | 0: > 85%  1: 75-84%  2: 65-74%  3: < 65% |  |
| Sleep Disturbances | Occurrence of any sleep disturbances (pain, urge to urinate, bad dreams, etc.) and their frequency | **Sum of all questions b-j (0-3):**  Not during the past month (0)  Less than once a week (1)  Once or twice a week (2)  Three or more times a week (3) |  |
| Use of Sleep Meds | Use of sleep medicine and its frequency | Not during the past month (0)  Less than once a week (1)  Once or twice a week (2)  Three or more times a week (3) |  |
| Daytime Dysfunction | (1) Troubles with staying awake during driving, eating or engaging in social activity  (2) Problem with keeping up enough enthusiasm to get things done | **Sum of both questions (0-3):**  Not during the past month (0)  Less than once a week (1)  Once or twice a week (2)  Three or more times a week (3)  No problem at all (0)  Only a very slight problem (1)  Somewhat of a problem (2)  A very big problem (3) |  |

**Supplementary Material 2: Neurocognitive Tests in the HCP sample**

| **Test** | **Subdomain** | **Description** | **Score** | **Reference** |
| --- | --- | --- | --- | --- |
| NIH Toolbox Picture Sequence Memory | non-verbal episodic memory | The subject is presented a particularly ordered series of illustrated activities and objects increasing in length and recalls this order while being shown the same pictures disorderedly. | Total count of correct pairs of adjacent pictures | [Weintraub et al., 2013] |
| NIH Toolbox Dimensional Change Card Sort Test | executive function, cognitive flexibility | The subject matches bivalent cards to one of two target cards following either shape or color. The rule switches after several trials. | Factors in accuracy and reaction time | [Weintraub et al., 2013] |
| NIH Toolbox Flanker Inhibitory Control and Attention Test | executive function | The subject indicates the direction of the central one of three arrows. | Factors in accuracy and reaction time | [Weintraub et al., 2013] |
| Penn Progressive Matrices, total correct responses | fluid intelligence | The subject is shown matrices of 2x2, 3x3 or 1x5 squares containing one absent square and selects the most suitable out of five respond options to fill the gap. | Number of correct answers | [Bilker et al., 2012] |
| NIH Toolbox Oral Reading Recognition Test | reading decoding skills | The subject reads and articulates letters and words as precise as possible, while the examiner compares the response with a list of accepted pronunciations. Computer adaptive testing performs item selection. | Higher precision results in a higher score. | [Weintraub et al., 2013] |
| NIH Toolbox Picture Vocabulary Test | vocabulary knowledge | The subject listens to a word and picks the corresponding picture out of four response choices. Computer adaptive testing performs item selection. | A higher score displays larger vocabulary knowledge. | [Weintraub et al., 2013] |
| NIH Toolbox Pattern Comparison Processing Speed Test | processing speed | The subject must indicate whether two uncomplicated images, presented next to each other, are equal. | Number of correct answers given within 90 seconds | [Weintraub et al., 2013] |
| Delay Discounting | self-regulation/ Impulsivity | The subject chooses between a fixed delayed reward and a variable, smaller immediate amount. The first trial starts with half the delayed reward. Depending on the subject’s choice the amount is adjusted after each trial to approximate a point of equivalence after 5 trials for every combination of reward and delay. | Area-under-the-curve measure summarizing all trials for 40000$ delayed reward | [Estle et al., 2006; Green et al., 2007; Myerson et al., 2001] |
| Variable Short Penn Line Orientation | spatial orientation processing | The subject parallelizes two angled lines with a certain distance by rotating one of the lines. | Number of correct items | [Gur et al., 2001; Gur et al., 2010] |
| Short Penn Continuous Performance Test | sustained attention | The subject is shown vertical and horizontal lines transiently and responds, if the lines shape a number or a letter. | Specificity of right decisions | [Gur et al., 2001; Gur et al., 2010] |
| Penn Word Memory Test | verbal episodic memory | The subject tries to memorize 20 written words and to consecutively identify the previously seen words out of 40 words (the known 20 and 20 distractors). | Number of correct answers | [Gur et al., 2001; Gur et al., 2010] |
| NIH Toolbox List Sorting Working Memory Test | working memory | The subject sorts different visually or auditorily presented items by size. | Number of correct items | [Weintraub et al., 2013] |

**Supplementary Material 3: Regression analyses for quadratic associations between sleep duration, global cognition and white matter microstructure**

Methods:

To test whether there is an inverse U-shaped association between sleep duration on the one hand and cognition and FA on the other, two quadratic regressions (1. global cognition score as dependent variable; 2. FA as dependent variable) with a squared sleep duration regressor (beside sleep duration, age and sex) in each model were estimated.

Results:

The quadratic regression model with global cognition as the dependent variable yielded a significant effect of the coefficient on the squared sleep duration regressor (ß = -.577; p = .001, for full regression analysis see below), suggesting an inverse U-shaped relationship of sleep duration and cognitive performance (see Supplementary Figure 1 for illustration). The maximum level of cognition was reached at a sleep duration of 7.73 hours (7 hours 44 minutes; see below) with cognition levels declining for sleep durations beyond that. However, the linear sleep duration regressor remained significant in this model as well (ß = .697; p < .001; see Supplementary Figure 2 for illustration). Additionally, the specification of a quadratic regression model revealed no significant association of coefficient of the squared sleep duration regressor with fractional anisotropy (ß = .139, p = .527; please see below).

1. Regression Analysis: nonlinear association Sleep-FA

| **Coefficients^a^** | | | | | | |
| --- | --- | --- | --- | --- | --- | --- |
| Model | | Unstandardized Coefficients | | Standardized Coefficients | t | Sig. |
|  |  | B | Std. Error | Beta |  |  |
| 1 | (Constant) | ,611 | ,023 |  | 26,602 | ,000 |
|  | Sleep duration squared | ,000 | ,001 | ,139 | ,632 | ,527 |
|  | Sleep duration | ,000 | ,007 | -,009 | -,040 | ,968 |
|  | Age | 9,460E-5 | ,000 | ,010 | ,323 | ,747 |
|  | Gender | ,004 | ,002 | ,061 | 1,960 | ,050 |
| a. Dependent Variable: Mean Fractional Anisotropy from Superior Longitudinal Fasciscles | | | | | | |

1. Regression Analysis: nonlinear association Sleep-Cognition

| **Coefficients^a^** | | | | | | |
| --- | --- | --- | --- | --- | --- | --- |
| Model | | Unstandardized Coefficients | | Standardized Coefficients | t | Sig. |
|  |  | B | Std. Error | Beta |  |  |
| 1 | (Constant) | 98,389 | 8,431 |  | 11,670 | ,000 |
|  | Sleep duration squared | -,577 | ,174 | -,608 | -3,309 | **,001** |
|  | Sleep duration | 8,926 | 2,353 | ,697 | 3,794 | **,000** |
|  | Age | -,202 | ,118 | -,051 | -1,721 | ,085 |
|  | Gender | -2,969 | ,868 | -,100 | -3,423 | ,001 |
| a. Dependent Variable: Global cognition score  see Supplementary Figure 1 and 2 below for scatterplots of the quadratic and linear associations. | | | | | | |

1. Calculation of the local maximum of sleep duration-cognition association

In order to derive the value of sleep duration that represents the turning point, i.e. the local maximum, with respect to cognition levels reached, we derive the first order condition of the regression function:

$$\hat{y}_{i}=ß_{0i}+ ß_{1i}*{{Sleep duration}^{2}}_{i}+ ß_{2i}*{Sleep duration}_{2i}+\ldots+u_{i}$$

$$\frac{\partial\hat{y}}{\partial sleep duration}=-0.577*2*Sleep duration+ 8.926 = 0$$

$$7.7348=Sleep duration$$

Local maximum sleep duration-cognition association: 7.73 hours = 7 hours 44min

**Supplementary Figure 1. Scatterplot of nonlinear (quadratic) analysis: Inverse U-shaped association of sleep duration and cognition**


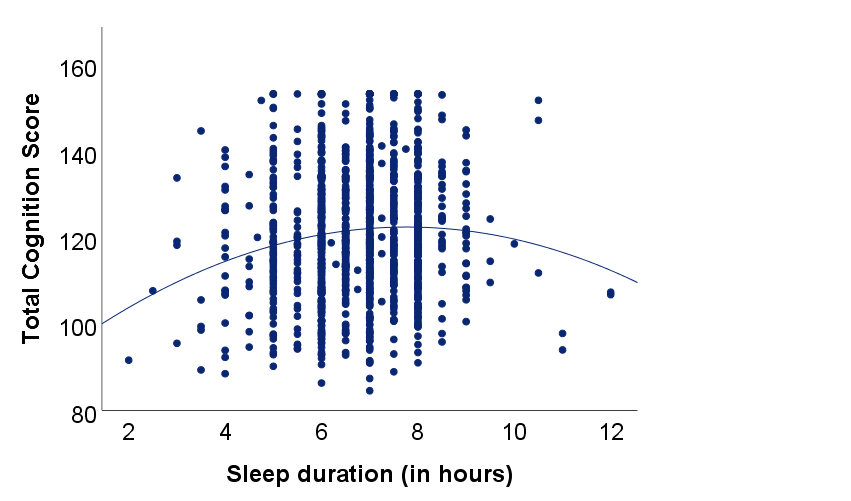


**Supplementary Figure 2. Scatterplot of linear analysis: Positive association of sleep duration and cognition**


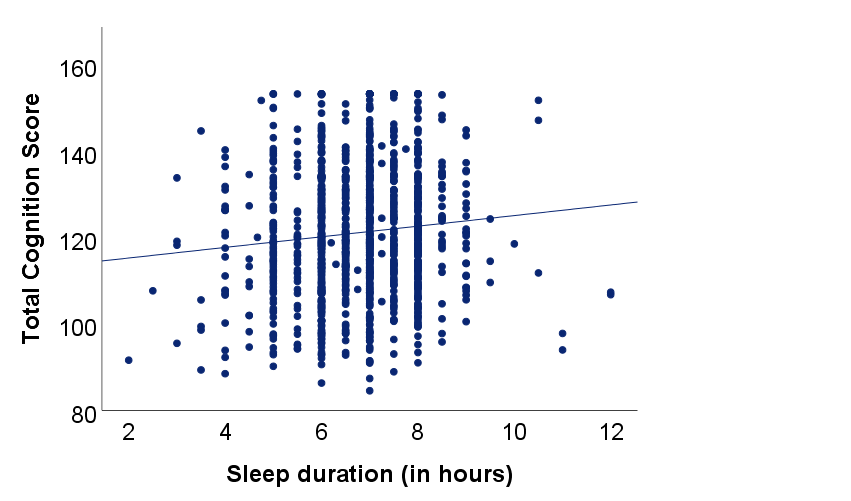


**Supplementary Material 4: Associations of short, moderate and prolonged sleep duration with cognitive performance and fractional anisotropy**

Methods: To further investigate effects, we divided our sample into three subsamples based on sleep duration [Watson et al., 2015] (short 0-5h, n = 107; moderate 5.5-8h, n = 882; prolonged 8.5-12h, n = 55) and correlated sleep duration in each subgroup with FA and cognition.

Results: With regard to the global cognition score, for short sleep duration there remains a trend-level significance for a positive association (r = .161; p = .097; df = 104), moderate sleep duration positively correlates with global cognition (r = .074; p = .028; df = 880) and prolonged sleep duration was negatively associated (r = -.393; p = .003; df = 53; see Table below for complete results).

Supplementary Table: Associations of short, moderate and prolonged sleep duration with cognitive performance and fractional anisotropy

|  | | Short Sleep Duration | Moderate Sleep Duration | Prolonged Sleep Duration |
| --- | --- | --- | --- | --- |
| NIH Total Cognition Score | Pearson's r | .161 | **.074^*^** | **-.393^**^** |
|  | p-value | .097 | **.028** | **.003** |
|  | df | 105 | **880** | **53** |
| Flanker Inhibitory Control and Attention Test:  executive function | Pearson's r | .088 | -.010 | -.230 |
|  | p-value | .366 | .759 | .091 |
|  | df | 105 | 880 | 53 |
| Picture Sequence Memory:  [non-verbal] episodic memory | Pearson's r | .153 | .058 | -.236 |
|  | p-value | .117 | .085 | .082 |
|  | df | 105 | 880 | 53 |
| List Sorting Working Memory Test | Pearson's r | .263 | **.072^*^** | **-.423^**^** |
|  | p-value | .006 | **.033** | **.001** |
|  | df | 105 | **880** | **53** |
| Picture Vocabulary Test:  vocabulary knowledge | Pearson's r | .132 | .058 | **-.320^*^** |
|  | p-value | .174 | .087 | **.017** |
|  | df | 105 | 880 | **53** |
| Oral Reading Recognition Test:  reading decoding skills | Pearson's r | .100 | **.099^*^** | -.234 |
|  | p-value | .303 | **.003** | .085 |
|  | df | 105 | **880** | 53 |
| Dimensional Change Card Sort Test:  executive function & cognitive flexibility | Pearson's r | .006 | .008 | -.105 |
|  | p-value | .951 | .808 | .447 |
|  | df | 105 | 880 | 53 |
| Pattern Comparison Processing Speed Test | Pearson's r | .050 | .008 | -.186 |
|  | p-value | .608 | .803 | .174 |
|  | df | 105 | 880 | 53 |
| Fractional Anisotropy in the SLF | Pearson's r | -.041 | **.089^**^** | -.001 |
|  | p-value | .676 | **.008** | .995 |
|  | df | 105 | **880** | 53 |

Short Sleep Duration: 0-5 hours of actual sleep at night; Moderate Sleep Duration: 5.5-8 hours of actual sleep at night; Prolonged Sleep Duration: 8.5-12 hours of actual sleep at night; SLF: Superior longitudinal fasciculus; FA: Fractional anisotropy, mean value extracted from the FA-global cognition association results mask; Pearson’s r = Pearson correlation coefficient; p-value= value determining statistical significance, *** p < .001; ** p < 0.01; * p < 0.05, values < .05 in bold font; df = degrees of freedom; for more information on the cognitive subscores see Supplementary Material 2.

**Supplementary Material 5: Associations of BMI with sleep and cognition scales**

|  | | BMI**^1^** |
| --- | --- | --- |
| NIH Total Cognition Score | Pearson's r | **-.187^***^** |
|  | p-value | **<.001** |
|  | df | **1182** |
| Flanker Inhibitory Control and Attention Test:  executive function | Pearson's r | **-.057^*^** |
|  | p-value | **.048** |
|  | df | **1182** |
| Picture Sequence Memory:  [non-verbal] episodic memory | Pearson's r | **-.089^**^** |
|  | p-value | **.002** |
|  | df | **1182** |
| List Sorting Working Memory Test | Pearson's r | -.056 |
|  | p-value | .056 |
|  | df | 1182 |
| Picture Vocabulary Test:  vocabulary knowledge | Pearson's r | **-.176^***^** |
|  | p-value | **<.001** |
|  | df | **1182** |
| Oral Reading Recognition Test:  reading decoding skills | Pearson's r | **-.212^***^** |
|  | p-value | **<.001** |
|  | df | **1182** |
| Dimensional Change Card Sort Test: executive function & cognitive flexibility | Pearson's r | **-.124^***^** |
|  | p-value | **<.001** |
|  | df | **1182** |
| Pattern Comparison Processing Speed Test | Pearson's r | **-.073^*^** |
|  | p-value | **.012** |
|  | df | **1182** |

|  | | BMI |
| --- | --- | --- |
| Global PSQI score | Spearman's Rho | **.094^**^** |
|  | p-value | **.001** |
|  | n | **1205** |
| Subjective Sleep Quality | Spearman's Rho | **.072^*^** |
|  | p-value | **.012** |
|  | n | **1205** |
| Sleep Latency | Spearman's Rho | .054 |
|  | p-value | .061 |
|  | n | 1205 |
| Sleep Duration | Spearman's Rho | **.131^***^** |
|  | p-value | **<.001** |
|  | n | **1205** |
| Habitual Sleep Efficiency | Spearman's Rho | **.078^**^** |
|  | p-value | **.006** |
|  | n | **1205** |
| Sleep Disturbance | Spearman's Rho | **.082^**^** |
|  | p-value | **.004** |
|  | n | **1205** |
| Use of Sleep Meds | Spearman's Rho | -.034 |
|  | p-value | .233 |
|  |  | 1205 |
| Daytime Dysfunction | Spearman's Rho | .027 |
|  | p-value | .351 |
|  | n | 1205 |

BMI = Body Mass Index (mass in kg/square of the body height in m^2^); Pearson’s r = Pearson correlation coefficient; Spearman's Rho = Spearman correlation coefficient; p-value= value determining statistical significance, *** p < .001; ** p < 0.01; * p < 0.05, values < .05 in bold font; df = degrees of freedom; n= number of subjects available for this distinct analysis; for description of all PSQI subcomponents, please see Supplementary Material 1; for more information on the cognitive subscores see Supplementary Material 2.

**^1^** Controlling for age and sex

**Supplementary Material 6: Correlation of Sleep Duration and Fractional Anisotropy with Cognitive Subscores controlling for sex, age and BMI**

|  | | Sleep Duration | Fractional Anisotropy within SLF |
| --- | --- | --- | --- |
| NIH Total Cognition Score | Pearson's r | **.085^**^** | **.085^**^** |
|  | p-value | **.003** | **.006** |
|  | df | **1181** | **1044** |
| Flanker Inhibitory Control and Attention Test:  executive function | Pearson's r | .011 | .008 |
|  | p-value | .699 | .794 |
|  | df | 1181 | 1044 |
| Picture Sequence Memory:  [non-verbal] episodic memory | Pearson's r | .046 | **.062^*^** |
|  | p-value | .116 | **.045** |
|  | df | 1181 | **1044** |
| List Sorting Working Memory Test | Pearson's r | .037 | **.086^**^** |
|  | p-value | .207 | **.005** |
|  | df | 1181 | **1044** |
| Picture Vocabulary Test:  vocabulary knowledge | Pearson's r | **.105^***^** | .033 |
|  | p-value | **<.001** | .279 |
|  | df | **1181** | 1044 |
| Oral Reading Recognition Test:  reading decoding skills | Pearson's r | **.122^***^** | .055 |
|  | p-value | **<.001** | .076 |
|  | df | **1181** | 1044 |
| Dimensional Change Card Sort Test:  executive function & cognitive flexibility | Pearson's r | .040 | .052 |
|  | p-value | .167 | .094 |
|  | df | 1181 | 1044 |
| Pattern Comparison Processing Speed Test | Pearson's r | -.009 | .052 |
|  | p-value | .765 | .092 |
|  | df | 1181 | 1044 |

Sleep duration: Hours of actual sleep at night; SLF: Superior longitudinal fasciculus; FA: Fractional anisotropy, mean value extracted from the FA-global cognition association results mask; BMI = Body Mass Index (mass in kg/square of the body height in m^2^); Pearson’s r = Pearson correlation coefficient; p-value= value determining statistical significance, *** p < .001; ** p < 0.01; * p < 0.05, values < .05 in bold font; df = degrees of freedom; for more information on the cognitive subscores see Supplementary Material 2.

|  | | Global PSQI Score | Subjective Sleep Quality | Sleep Latency | Sleep Duration | Habitual Sleep Efficiency | Sleep Disturbance | Use of Sleep Meds | Daytime Dysfunction |
| --- | --- | --- | --- | --- | --- | --- | --- | --- | --- |
| Global PSQI Score | Spearman's Rho | 1 | **.667^***^** | **.588^***^** | **.575^***^** | **.571^***^** | **.495^***^** | **.345^***^** | **.455^***^** |
|  | p-value |  | **<.001** | **<.001** | **<.001** | **<.001** | **<.001** | **<.001** | **<.001** |
| Subjective Sleep Quality | Spearman's Rho |  | 1 | **.310^***^** | **.345^***^** | **.269^***^** | **.321^***^** | **.142^***^** | **.245^***^** |
|  | p-value |  |  | **<.001** | **<.001** | **<.001** | **<.001** | **<.001^***^** | **<.001** |
| Sleep Latency | Spearman's Rho |  |  | 1 | **.101^***^** | **.202^***^** | **.293^***^** | **.203^***^** | **.105^***^** |
|  | p-value |  |  |  | **<.001** | **<.001** | **<.001** | **<.001** | **<.001** |
| Sleep Duration | Spearman's Rho |  |  |  | 1 | **.391^***^** | **.109^***^** | .014 | **.151^***^** |
|  | p-value |  |  |  |  | **<.001** | **<.001** | .623 | **<.001** |
| Habitual Sleep Efficiency | Spearman's Rho |  |  |  |  | 1 | **.193^***^** | **.081^**^** | **.099^**^** |
|  | p-value |  |  |  |  |  | **<.001** | **.005** | **.001** |
| Sleep Disturbance | Spearman's Rho |  |  |  |  |  | 1 | **.102^***^** | **.217^***^** |
|  | p-value |  |  |  |  |  |  | **<.001** | **<.001** |
| Use of Sleep Meds | Spearman's Rho |  |  |  |  |  |  | 1 | .043 |
|  | p-value |  |  |  |  |  |  |  | .137 |
| Daytime Dysfunction | Spearman's Rho |  |  |  |  |  |  |  | 1 |
|  | p-value |  |  |  |  |  |  |  |  |

**Supplementary Material 7: Correlation matrix of all PSQI Subcomponents**

Global PSQI Score: reflects the overall sleep quality on a 0-21 scale and consist of seven subcomponents; Spearman's Rho = Spearman correlation coefficient; p-value = value determining statistical significance, *** p < .001; ** p < 0.01; * p < 0.05, values < .05 in bold font; n = 1206 for all correlations; for description of all PSQI subcomponents, please see Supplementary Material 1.

**Supplementary Material 8: Results for non-parametric tests of PSQI subscores and cognitive performance**

|  | | Global PSQI Score | Sleep Latency | Habitual Sleep Efficiency | Sleep Dis-turbance | Use of Sleep Meds | Daytime Dys-function | Sleep Duration | Sleep Quality |
| --- | --- | --- | --- | --- | --- | --- | --- | --- | --- |
| NIH Total Cognition Score | Spearman’s Rho | **-.102^***^** | **-.109^***^** | **-.062^*^** | **-.108^***^** | -.006 | .047 | **-.114^***^** | .006 |
|  | p-value | **<.001** | **<.001** | **.031** | **<.001** | .828 | .104 | **<.001** | .424 |
|  | n | **1187** | **1187** | **1187** | **1187** | 1187 | 1187 | **1187** | 1187 |
| Flanker Inhibitory Control and Attention Test:  executive function | Spearman's Rho | -.033 | -.043 | -.004 | **-.066^*^** | -.041 | -.017 | -.012 | -.005 |
|  | p-value | .256 | .139 | .895 | **.021** | .159 | .565 | .680 | .436 |
|  | n | 1206 | 1206 | 1206 | **1206** | 1206 | 1206 | 1206 | 1206 |
| Picture Sequence Memory:  [non-verbal] episodic memory | Spearman's Rho | -.033 | -.042 | .052 | -.010 | -.044 | .052 | **-.068^*^** | -.003 |
|  | p-value | .258 | .145 | .073 | .727 | .129 | .074 | **.018** | .465 |
|  | n | 1205 | 1205 | 1205 | 1205 | 1205 | 1205 | **1205** | 1205 |
| List Sorting Working Memory Test | Spearman's Rho | -.032 | -.051 | .008 | -.030 | -.015 | -.004 | -.052 | .033 |
|  | p-value | .270 | .074 | .792 | .304 | .607 | .887 | .074 | .129 |
|  | n | 1206 | 1206 | 1206 | 1206 | 1206 | 1206 | 1206 | 1206 |
| Picture Vocabulary Test:  vocabulary knowledge | Spearman's Rho | **-.123^***^** | **-.117^***^** | **-.153^***^** | **-.099^**^** | .027 | .053 | **-.124^***^** | .008 |
|  | p-value | **<.001** | **<.001** | **<.001** | **.001** | .352 | .064 | **<.001** | .386 |
|  | n | **1206** | **1206** | **1206** | **1206** | 1206 | 1206 | **1206** | 1206 |
| Oral Reading Recognition Test:  reading decoding skills | Spearman's Rho | **-.128^***^** | **-.133^***^** | **-.142^***^** | **-.124^***^** | .038 | .070* | **-.134^***^** | -.006 |
|  | p-value | **<.001** | **<.001** | **<.001** | **<.001** | .191 | .015 | **<.001** | .417 |
|  | n | **1206** | **1206** | **1206** | **1206** | 1206 | 1206 | **1206** | 1206 |
| Dimensional Change Card Sort Test:  executive function & cognitive flexibility | Spearman's Rho | -.041 | -.025 | -.026 | **-.068^*^** | -.011 | -.025 | -.040 | .012 |
|  | p-value | .157 | .382 | .369 | **.018** | .695 | .391 | .163 | .340 |
|  |  | 1203 | 1203 | 1203 | **1203** | 1203 | 1203 | 1203 | 1203 |
| Pattern Comparison Processing Speed Test | Spearman's Rho | -.036 | -.032 | .020 | **-.068^*^** | -.023 | -.004 | -.023 | -.020 |
|  | p-value | .206 | .269 | .492 | **.018** | .418 | .892 | .432 | .243 |
|  | n | 1206 | 1206 | 1206 | **1206** | 1206 | 1206 | 1206 | 1206 |
| Fractional anisotropy in the SLF | Spearman's Rho | **-.063^*^** | -.026 | -.023 | -.029 | -.051 | .005 | **-.112^***^** | -.006 |
|  | p-value | **.040** | .394 | .461 | .337 | .096 | .867 | **<.001** | .856 |
|  | n | **1065** | 1065 | 1065 | 1065 | 1065 | 1065 | **1065** | 1065 |

Global PSQI Score: reflects the overall sleep quality on a 0-21 scale and consist of seven subcomponents (higher scores indicate poorer sleep quality); FA: Fractional anisotropy, mean value extracted from the FA-sleep duration association results mask; SLF = superior longitudinal fascicle; Spearman's Rho = Spearman correlation coefficient; p-value = value determining statistical significance, *** p < .001; ** p < 0.01; * p < 0.05, values < .05 in bold font; n= number of subjects available for this distinct analysis;; for description of all PSQI subcomponents, please see Supplementary Material 1; for more information on the cognitive subscores see Supplementary Material 2.

**Supplementary Material 9: Age interaction analyses**

To investigate the role of age on the strength of the observed association of sleep duration with FA and cognition, we performed additional interaction analyses. To this end, we performed

1. an analyses of covariance (ANCOVA) with global cognition as the dependent variables and age, sex, sleep duration and a sleep duration* age interaction term as the covariates.
2. an ANCOVA with mean FA (from the SLF) as the dependent variables and age, sex, sleep duration and a sleep duration* age interaction term as the covariates.

Both analyses did not show a significant age*sleep duration interaction (p= .881 for the cognition analysis and p= .452 for the FA analysis).

**Supplementary Material 10: Trend-level Results for the FA-sleep duration association**

Here we report on the whole-brain trend-level (p< .099, FWE-corrected) positive association of sleep duration and FA

Supplementary Figure


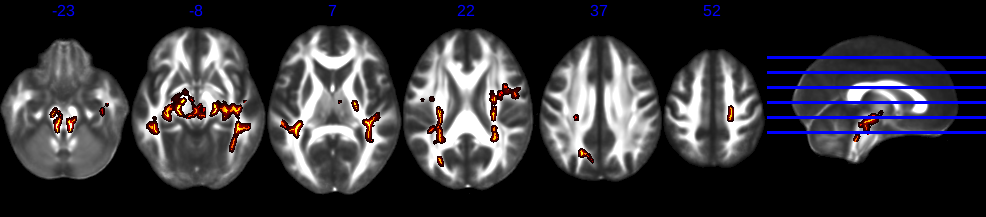


Positive association of Sleep duration and Fractional Anisotropy. Axial slices with corresponding y-axis values (MNI) are presented. Red-yellow areas represent voxels (using FSL’s „fill“ command for better visualization), where a significant positive association between sleep duration and Fractional Anisotropy was detected (pFWE < .099).

Cluster information

| **Cluster size (voxel):** | **Significance (1-p)** | **MNI x-value** | **MNI y-value** | **MNI z-value** |
| --- | --- | --- | --- | --- |
| 2840 | 0.951 | 4 | -20 | -14 |
| 2788 | 0.955 | 37 | -31 | 4 |
| 2234 | 0.936 | -42 | -36 | -9 |
| 393 | 0.920 | -26 | -70 | 22 |
| 299 | 0.910 | 43 | -3 | 26 |
| 176 | 0.912 | -35 | -9 | 24 |
| 162 | 0.905 | 27 | -24 | 20 |
| 128 | 0.915 | 19 | -25 | 52 |
| 62 | 0.901 | -5 | -8 | -10 |

Dimensions of clusters (number of voxels) and localization of signal peaks (MNI coordinates) are given for regions showing maximal effects of tract-based spatial statistics values (signal peak).

Table Tracts:

Unclassified:44.8249

Middle cerebellar peduncle:0.814798

Pontine crossing tract (a part of MCP):0.517507

Corticospinal tract R:0.110108

Corticospinal tract L:2.60956

Medial lemniscus R:1.32129

Medial lemniscus L:1.29927

Inferior cerebellar peduncle R :0.385378

Inferior cerebellar peduncle L:0.649637

Superior cerebellar peduncle R:1.49747

Superior cerebellar peduncle L:1.39837

Cerebral peduncle R:2.11407

Cerebral peduncle L:3.22616

Anterior limb of internal capsule R:0.0770755

Posterior limb of internal capsule R:3.061

Posterior limb of internal capsule L:0.506496

Retrolenticular part of internal capsule R:3.72165

Retrolenticular part of internal capsule L:3.28122

Superior corona radiata R:1.68465

Superior corona radiata L:0.880863

Posterior corona radiata R:2.77472

Posterior corona radiata L:2.87382

Posterior thalamic radiation (include optic radiation) R:4.54746

Posterior thalamic radiation (include optic radiation) L:1.18917

Sagittal stratum (include inferior longitudinal fasciculus and inferior fronto-occipital fasciculus) R:3.71064

Sagittal stratum (include inferior longitudinal fasciculus and inferior fronto-occipital fasciculus) L:1.70667

External capsule R:0.27527

Fornix (cres) / Stria terminalis (can not be resolved with current resolution) R:2.09205

Fornix (cres) / Stria terminalis (can not be resolved with current resolution) L:2.2462

Superior longitudinal fasciculus R:1.06805

Superior longitudinal fasciculus L:3.49042

Tapetum R:0.0440432

Reported are white matter tracts in the cluster based on the JHU ICBM-DTI-81 White-Matter Labels (as implemented in FSL). R= right; L= left; Probabilities of affected tracts: It gives the (average) probability of all significant voxels being a member of the different labelled regions within the atlas (JHU ICBM-DTI-81 White-Matter), calculated with the FSL tool “atlasquery”.

**References**

Bilker WB, Hansen JA, Brensinger CM, Richard J, Gur RE, Gur RC (2012): Development of Abbreviated Nine-Item Forms of the Raven’s Standard Progressive Matrices Test. Assessment.

Buysse, D.J., Reynolds, C.F., Monk, T.H., Berman, S.R., & Kupfer DJ (1989): The Pittsburgh Sleep Quality Index: A New Instrument for Psychiatric Practice and Research. Psychiatry Res 28:193–213.

Estle SJ, Green L, Myerson J, Holt DD (2006): Differential effects of amount on temporal and probability discounting of gains and losses. Mem Cogn.

Green L, Myerson J, Shah AK, Estle SJ, Holt DD (2007): Do Adjusting-Amount and Adjusting-Delay Procedures Produce Equivalent Estimates of Subjective Value in Pigeons? J Exp Anal Behav.

Gur RC, Ragland JD, Moberg PJ, Turner TH, Bilker WB, Kohler C, Siegel SJ, Gur RE (2001): Computerized neurocognitive scanning: I. Methodology and validation in healthy people. Neuropsychopharmacology.

Gur RC, Richard J, Hughett P, Calkins ME, Macy L, Bilker WB, Brensinger C, Gur RE (2010): A cognitive neuroscience-based computerized battery for efficient measurement of individual differences: Standardization and initial construct validation. J Neurosci Methods.

Myerson J, Green L, Warusawitharana M (2001): Area under the curve as a measure of discounting. J Exp Anal Behav 76:235–243.

Watson NF, Badr MS, Belenky G, Bliwise DL, Buxton OM, Buysse D, Dinges DF, Gangwisch J, Grandner MA, Kushida C, Malhotra RK, Martin JL, Patel SR, Quan SF, Tasali E, Twery M, Croft JB, Maher E, Barrett JA, Thomas SM, Heald JL (2015): Joint Consensus Statement of the American Academy of Sleep Medicine and Sleep Research Society on the Recommended Amount of Sleep for a Healthy Adult: Methodology and Discussion. J Clin Sleep Med 11:931–952.

Weintraub S, Dikmen SS, Heaton RK, Tulsky DS, Zelazo PD, Bauer PJ, Carlozzi NE, Slotkin J, Blitz D, Wallner-Allen K, Fox NA, Beaumont JL, Mungas D, Nowinski CJ, Richler J, Deocampo JA, Anderson JE, Manly JJ, Borosh B, Havlik R, Conway K, Edwards E, Freund L, King JW, Moy C, Witt E, Gershon RC (2013): Cognition assessment using the NIH Toolbox. Neurology 80:S49–S53.
